# A Neonate with an Unusual Midline Defect and Cardiovascular Anomaly

**DOI:** 10.1055/s-0037-1612619

**Published:** 2018-01-22

**Authors:** Martin Poryo, Paul Hoffmann, Hans-Joachim Schäfers, Clemens-Magnus Meier, Katrin Altmeyer, Hashim Abdul-Khaliq, Michael Zemlin, Sascha Meyer

**Affiliations:** 1 Department of Pediatric Cardiology, Saarland University Medical Center, Homburg/Saar, Germany; 2 Department of Pediatrics and Neonatology, Saarland University Medical Center, Homburg/Saar, Germany; 3 Department of Thoracic and Cardiovascular Surgery, Saarland University Medical Center, Homburg/Saar, Germany; 4 Department of General, Visceral, Vascular and Pediatric Surgery, Saarland University Medical Center, Homburg/Saar, Germany; 5 Department of Diagnostic and Interventional Radiology, Saarland University Medical Center, Homburg/Saar, Germany

**Keywords:** aortic aneurysm, sternal cleft, surgical repair

## Abstract

We present a female neonate with a sternal cleft (SC) and additional aortic aneurysm who presented with respiratory failure. Stabilization of the SC was achieved by using the xyphoid process as an autologous graft bridging the upper part of the SC. We conclude that a step-wise correction of the SC with the use of an autologous graft may improve respiratory function, and should be considered when complete surgical correction is not feasible.

## Introduction

 The occurrence of a sternal cleft (SC) is very rare in children, 1 2 and may be associated with several clinical problems; 3 most importantly, respiratory dysfunction secondary to disturbances in respiratory mechanics. Only a very few reports have been published that reported a combination of SC with aortic aneurysms (AA). 4 5 6 7 8

Here, we present a female neonate with an AA who underwent SC repair.

## Case Report

We report on a term female newborn with a prenatally diagnosed isolated fetal aneurysm of the ascending aorta, but without further organ-specific diagnostic workup. As there was no evidence for an infection in this child, TORCH serology was not performed as per protocol of our center.

**Fig. 1:**
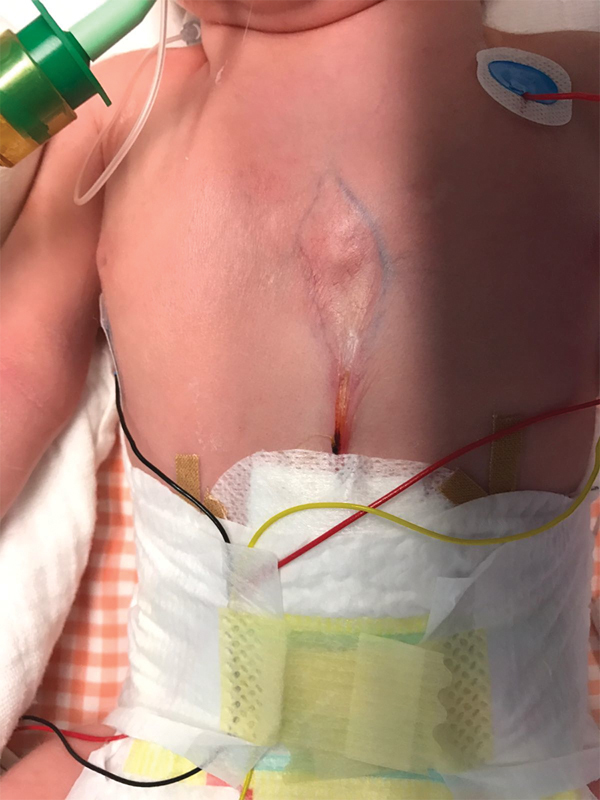
Presentation of the strand with extension from the xiphoid process to the umbilical cord.

 Initial cardiopulmonary adaption was normal (APGAR scores of 8/9/9 at 1, 5, and 10 minutes). Physical examination revealed a SC with a palpable, short osseous bridge near the xiphoid process (XP) with visualization of the pulsating aorta [ Video 1 (online only)]. Moreover, a reddish, padded strand extended from the XP to the umbilical cord ( Fig. 1 ). Otherwise, the physical examination was unrevealing. Due to secondary onset of respiratory insufficiency within the first hour of life, the girl was intubated and ventilated; attempts for extubation failed because of the instability of the anterior chest wall. 

**Video 1** Pulsating ascending aorta under the skin. Online content including video sequences viewable at: www.thieme-connect.com/products/ejournals/html/10-1055-s-0037-1612619-EJPSR-17-0327cg-v2.mp4 .

In the following diagnostic workup, echocardiography and CT [ Supplementary Fig. S1 (online only)] confirmed the diagnosis of an aneurysm of the ascending aorta (diameter of 14 mm, z-score +4.75) and SC. Genetic analysis was negative for the examined 25 genes causing Marfan syndrome and other thoracic AA and aortic dissection related-syndromes as well as Ehlers–Danlos syndrome: ACTA2, CBS, COL1A1, COL1A2, COL3A1, COL5A1, COL5A1, EFEMP2, FBN1, FLNA, MAT2A, MFAP5, MYH11, MYLK, NOTCH1, PLOD1, PRKG1, SKI, SLC2A10, SMAD3, TGFB2, TGFB3, TGFBR1, TGFBR2, TNXB. 

**Fig. 2:**
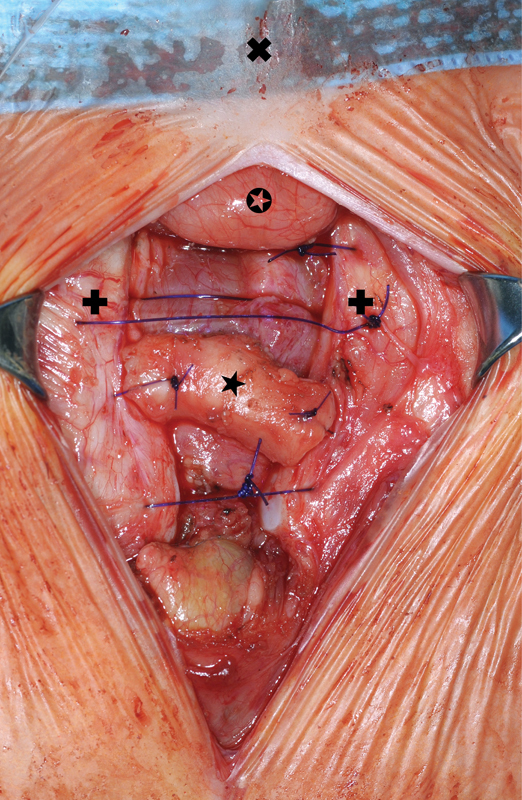
Intraoperative situs with autologous graft in the upper part of the SC (  cranial,  thymus,  sternum, ★ autologous graft).

 On the day 6 of life, staged surgical closure of the SC was staged by our thoracic and pediatric surgeons. Intraoperatively, the cartilage bridge including the XP was resected. Afterward, the SC was approximated by sutures [3–0 monofilament absorbable suture (Polydioxanone, PDS II, Ethicon, Somerville, NJ United States)]. By this maneuver, the lower two-thirds of the cleft were approximated. The removed part of the lower sternum including the XP was used as an autologous graft to close the upper part of the remaining cleft. In so doing, complete closure of the SC was achieved. However, immediately after closure of the cleft the neonate developed cardiopulmonary instability. Therefore, the XP was moved to the mid of the cleft and fixed by sutures (3–0 monofilament absorbable suture; Polydioxanone, PDS II, Ethicon, Somerville, NJ United States) to stabilize the thoracic anterior wall and to improve thoracic motility with no recurrence of cardiovascular instability ( Fig. 2 ). 

 The postoperative period was uneventful. The girl was self-ventilating on room air with only subtle recessions in the jugular fossa [ Video 2 (online only)]. The AA was not of relevance at this early stage, thus only medical treatment with β-blockers was initiated. The actual development of weight and growth is fine at the age of 6 months. There is no increased susceptibility for pulmonary infections, cardiac function is nearly normal, and the diameter of the ascending aorta decreased in size (now z-score of +2.6).

**Video 2** Recessions in the jugular fossa after extubation. Online content including video sequences viewable at: www.thieme-connect.com/products/ejournals/html/10-1055-s-0037-1604049-EJPSR-17-0322-CG-v1.mp4 .

## Discussion

 Successful surgical repair of SC by primary closure was first described by Maier and Bertone in 1949. 9 Several other surgical procedures have been reported since then including the use of costal cartilage grafts taken from the ribs, 10 autologous cartilage transfer, 11 or the bilateral sternal bar turnover flaps. 12 Also prosthetic materials like Teflon, silicone, or titanium have been used to close and stabilize the defect. 13

 In our patient, primary repair of the SC by direct approximation of the sternal bars as described by Shamberger and Welch 14 was planned. However, complete closure may not always be possible due to intraoperative onset of cardiocirculatory disturbances as in our patient. Thus, when complete surgical correction in SC is not feasible, the use of an autologous graft for stabilization should be considered. This can be seen as a bridging procedure, a first step of the correction of the SC and to gain time. Our case report demonstrates that stabilization of the anterior thoracic wall can improve respiratory mechanics with the restoration of adequate respiratory function. 

 Since this is an extremely rare case, there are no guidelines or recommendations regarding the correct time frame. Given the clinical course in our opinion, definitive closure of the SC in our patient should be postponed until the end of the first year of life. It can be expected that the cleft itself will not grow in size, thus when approximating both sternal bars, the intrathoracic pressure will not increase in the same manner as in the initial surgical procedure, and will be better tolerated. Permanent stabilization of the sternum can be achieved by a transsternally placed pectus bar or wires—comparable to surgical repair of pectus excavatum. 15

 The AA was not of relevance at this early stage and has demonstrated a relative decrease in size (z-score), thus only nonsurgical medical treatment (use of β-blockers to prevent further progression of the AA) was initiated as recommended in adult patients. 16

## Conclusion

A stepwise correction of a SC with the use of an autologous graft for stabilization may improve respiratory function and should be considered when complete surgical correction is not feasible, but complete closure of the SC should be realized early in infancy when the thoracic wall is still malleable.
