# Supplementary material for: A Neonate with an Unusual Midline Defect and Cardiovascular Anomaly
Source: European J Pediatr Surg Rep. 2018 Jan 22;6(1):e15–7. doi: 10.1055/s-0037-1612619 (PMC5786149; doi:10.1055/s-0037-1612619)
Supplement: Supplementary file 3 — Supplementary Figure [file 10-1055-s-0037-1612619-s170327cg-1.pdf]

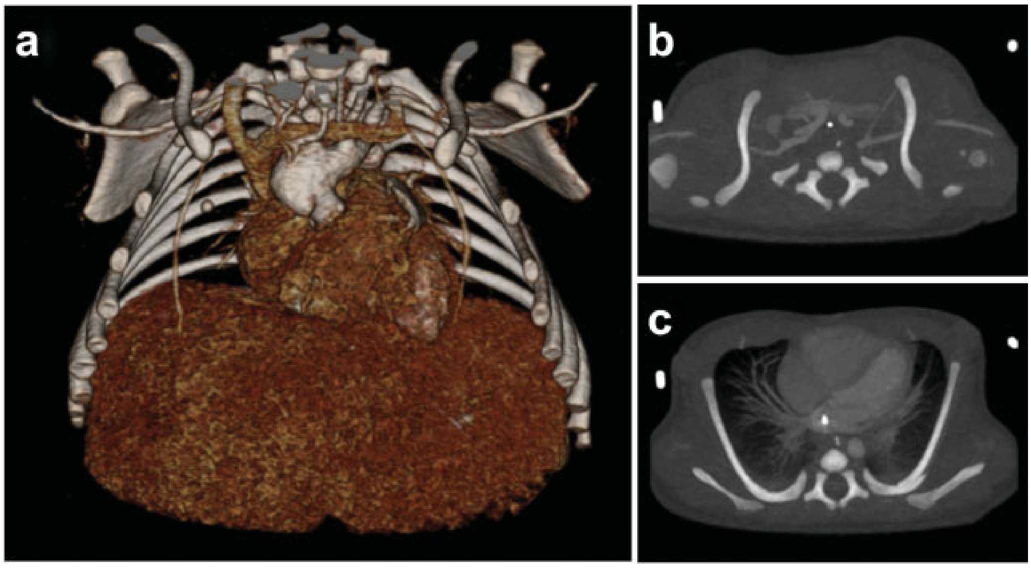

Fig. S1 ECG-gated CT angiography of the thoracic aorta. (**A**) Anterior volume-rendered projection shows anterior bulging of the enlarged ascending aorta as well as rib cage anomaly with lateral diverging of the anterior ribs. (**B**) Maximum intensity projection (MIP) at the level of the clavicles detects sagittal orientation of both clavicles. (**C**) Axial MIP aligned to the lateral ribs again displays thoracic deformity as well as subcutaneous position of the right ventricle due to sternal cleft.
